# Birth Weight in Type 1 Diabetic Pregnancy

**DOI:** 10.1155/2010/397623

**Published:** 2010-12-22

**Authors:** Jacquemyn Yves, Vandermotte Valerie, Van Hoorick Katrien, Martens Guy

**Affiliations:** ^1^Department of Obstetrics and Gynecology, Antwerp University Hospital UZA, Wilrijkstraat 10, 2650 Edegem, Belgium; ^2^Study Centre for Perinatal Epidemiology SPE, Hallepoortlaan, 1000 Brussel, Belgium

## Abstract

Our aim was to investigate whether birth weight in mothers with diabetes mellitus type 1 is higher as compared to nondiabetic controls. *Methods*. A retrospective study was performed using an existing database covering the region of Flanders, Belgium. Data included the presence of diabetes type 1, hypertension, parity, maternal age, the use artificial reproductive technology, fetal- neonatal death, congenital anomalies, admission to a neonatal intensive care unit, and delivery by Caesarean section or vaginally. *Results*. In the period studied, 354 women with diabetes type 1 gave birth and were compared with 177.471 controls. Women with type 1 diabetes more often had a maternal age of over 35 years (16.7% versus 12.0%, *P* = .008, OR 1.46; 95% CI 1.09–1.95). They more frequently suffered hypertension in pregnancy (19.5% versus 4.7%, *P* < .0001, OR 4.91; 95% CI 3.73–6.44). Perinatal death was significantly higher in the diabetes mellitus group (3.05% versus 0.73%, *P* < .0001, OR 4.28; 95% CI 2.22–8.01). Caesarean section was performed almost 5 times as frequently in the diabetes versus the control group (OR 4.57; 95% CI 3.70–5.65). Birth weight was significantly higher in diabetic pregnant women from 33 until 38 weeks included, but those reaching 39 weeks and later were not different with control groups. *Conclusion*. In Belgium, diabetic pregnancy still carries a high risk for fetal and maternal complications; in general birth weight is significantly higher but for those reaching term there is no significant difference in birth weight.

## 1. Introduction

The incidence of type 1 diabetes mellitus in Belgium varies between 10.9 to 15.4/100.000 [1]. In type 1 diabetic pregnancies, an increase in congenital malformations, miscarriage, macrosomia, shoulder dystocia, and other obstetric complications is described [2–19]. Macrosomia is defined as birth weight above the 90th percentile for gestational age. According to the modified Pederson hypothesis, maternal hyperglycemia results in transplacental diffusion of a higher amount of glucose to the fetus, resulting in a stimulation of the fetal beta cells and increased fetal insulin secretion. This finally results in fetal macrosomia. Macrosomia is related to obstetrical and neonatal complications such as asphyxia, shoulder dystocia, and neonatal trauma during delivery [6–9].

The aim of this study was to look for differences in birth weight in patients with diabetes type 1 as compared to the general obstetric population in the context of the actual therapy of diabetes.

## 2. Methods

A retrospective analysis was performed of an existing data base. The Study Centre for Perinatal Epidemiology registers data on all hospital deliveries (this is 99% of all deliveries) and most home deliveries in Flanders, Belgium. Data are collected on both maternal and neonatal outcome. The diagnosis of type 1 diabetes was accepted as given by the treating physician. For this study, hypertension was defined as a systolic blood pressure ≥140 mmHg and/or diastolic ≥90 mmHg; no further subdivision in pre-eclampsia, gestational hypertension, and pre-existent hypertension was made. Fetal death is death before delivery, early neonatal death is death in the first 28 days of live, and perinatal death was considered the sum of fetal and early neonatal death. For every fetus, the presence of congenital anomalies was registered as eventual transfer to a neonatal intensive care unit. Caesarean or vaginal delivery and the use of forceps or vacuum are also registered. Women with type 2 diabetes or gestational diabetes were excluded from our study. 

Dichotomic variables were compared using Chi squared test, significance accepted at *P* < .05, odds ratios, and 95% confidence intervals. To compare birthweight as a continuous variable per gestational week, a Shapiro-Wilk test was performed to test for normality; nonnormally distributed data were compared using the Mann-Whitney test, significance accepted at *P* < .05. Statistical analysis was done with SPSS 16.0.

## 3. Results

We studied patients who gave birth between January, 1st, 2002 and December, 1st 2004; during this period 354 women with type 1 diabetes gave birth and 177.407 control women. 

Table 1 gives a general overview of the obstetric data of our population. There were significantly more women in the type 1 diabetes group over age 35 years and significantly more maternal hypertension.  Table 2 demonstrates the fetal-neonatal outcome. Clearly fetal and perinatal deaths are significantly higher in the type 1 diabetes group as compared to controls, and significantly more congenital anomalies were seen in neonates from diabetic mothers. This correlates with more transfers to a neonatal intensive care unit. Neonates were almost 5 times as often born by Caesarean section in case of maternal diabetes type 1. Babies of mothers with diabetes type 1 were also more often preterm.

**Figure 1:**
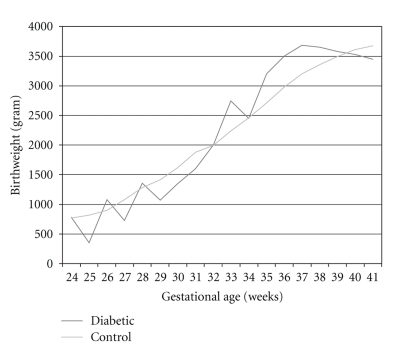
Median birthweight versus gestational age.

Table 3 presents birth weight per gestational week for diabetes type 1 and the control group.  Figure 1 presents a graphic view of the median birth weight per gestational week in diabetic and nondiabetic pregnancies. 

For none of the gestational weeks birthweight was normally distributed (probably due to low numbers), as the number of deliveries in the type 1 diabetic group was extremely low before 33 weeks (less than 5), we only compared median birthweight from week 33 till week 41. This demonstrates, as shown in Table 3, that until 38 weeks there is a significant difference in birth weight for the diabetic pregnancies; from 39 weeks on no significant difference is present.

## 4. Discussion

Although a higher birth weight in type 1 diabetes is generally accepted, only few studies have actually described birth weight per gestational week in diabetic pregnancies as compared to nondiabetic controls. Most studies give mean birth weights, not specifying gestational age. Lauszus et al. [13] described birth weight but made a division only in term and preterm pregnancies not allowing to determine whether a difference is or is not present at different gestational ages. 

Sibai et al. [14] compared birthweight in pregestational diabetes, chronic hypertension, and uncomplicated pregnancies before 35 weeks and between 35 and 37 weeks and noted a higher birthweight in pregestational diabetes. Dos Santos Silva et al. [19] made a very raw division in less than 33 weeks, between 33 and 36 weeks and more than 36 weeks but did not use a control group. 

The only study comparable to our present one we were able to find was that by Jensen et al. [10] who compared mean birth weight in the diabetic group versus a control group and did not find a significant difference. Evers et al. [16, 17] also used a control group but did not provide data per gestational week. As far as we know this is the first nationwide study presenting data on birth weight per gestational week in pregnancies with type 1 diabetes versus nondiabetic controls. Our data confirm that neonates from mothers with type 1 diabetes have a significantly increased risk for perinatal death, are more frequently born by Caesarean section, have more congenital anomalies, and more frequently need neonatal intensive care. As the number of babies born from type 1 diabetic mothers below 33 weeks was extremely low, it seemed not relevant to make a statistical comparison. When analyzing data between 33 and 41 weeks a significant difference was seen until 38 weeks. From 39 weeks on, there was no significant difference in birth weight between both groups. This can be due to chance, but another explanation might be that those pregnancies with type 1 diabetes that continue until 39 and more weeks are the ones with the least problems; all others will have been induced in the mean time.

Our study has several important limitations. We do not have any data on the quality of the diabetic control of our patients, we cannot also control for maternal weight or maternal weight gain, nor can we exclude the influence of other risk factors such as maternal smoking.

## 5. Conclusion

This retrospective analysis demonstrates that birth weight for neonates from mothers with type 1 diabetes is higher than that in a control group but the difference disappears at 39 weeks, probably because only the best regulated diabetic mothers continued their pregnancy until that week.

## Figures and Tables

**Table 1:** Maternal data.

	Type 1	%	Control	%	*P* value	Odds ratio	95% Cl
*N*	354		177471				
Maternal age ≥ 35 years	59	16.7	21362	12.0	.008	1.46	[1.09–1.95]
Parity = 1	179	50.6	83711	47.1	.201	1.15	[0.93–1.42]
Parity > 1	175	49.4	93760	52.8	.201	0.87	[0.70–1.08]
Hypertension	69	19.5	8346	4.7	.000	4.91	[3.73–6.44]
IVF	7	2.0	3779	2.1	.843	0.93	[0.40–2.02]
Hormonal stimulation	11	3.1	3489	2.0	.122	1.60	[0.83–2.99]

IVF: in vitro fertlisation. CI: Confidence interval.

**Table 2:** Fetal-neonatal outcome.

	type 1	%	Control	%	*P* value	Odds ratio	95% CI
*N*	361		180842				
Fetal death	9	2.49	923	0.51	.000	4.98	[2.40–9.96]
Perinatal death	11	3.05	1319	0.73	.000	4.28	[2.22–8.01]
Early neonatal death	2	0.57	396	0.22	.165	2.59	—
Late neonatal death	2	0.57	396	0.22	.174	2.54	—
Congenital abnormalities	21	5.80	3017	1.70	.000	3.64	[2.28–5.76]
NICU	80	22.20	7616	4.20	.000	6.48	[5.01–8.37]
Cesarean section	184	51.00	33490	18.50	.000	4.57	[3.70–5.65]
Forceps + ventouse	42	11.60	19738	10.90	.661	1.07	[0.77–1.50]
<37 w	98	27.7	13069	7.4	.000	4.82	[3.79–6.12]
<34 w	26	7.3	3350	1.9	.000	4.12	[2.70–6.24]
<28 w	5	1.4	765	0.4	.000	3.31	[1.21–8.30]

NICU: neonatal intensive care unit. CI: Confidence interval.

**Table 3:** Birth weight per gestational week.

Gestational week	Diabetes type 1		Control		*P* (Mann-Whitney
	Deliveries (=*N*)	Median birthweight (*g*)	Deliveries (=*N*)	Median birthweight (*g*)	
24	1	780	143	770	—
25	1	650	184	820	—
26	2	1075	231	900	—
27	1	723	270	1079	—
28	3	1352	263	1280	—
29	5	1070	376	1410	—
30	2	1350	467	1618	—
31	3	1600	730	1880	—
32	3	2000	1036	1992	—
33	8	2745	1843	2240	<.001
34	8	2448	2999	2460	<.001
35	20	3200	6218	2710	<.001
36	48	3505	13391	2980	<.001
37	91	3680	32196	3200	<.001
38	113	3650	47517	3360	<.001
39	35	3575	54628	3500	.076
40	12	3525	16955	3610	.856
41	5	3450	1022	3679	.627
